# Prevalence of Congenital Anomalies in Iran: A Review Article

**Published:** 2017-06

**Authors:** Soudabeh VATANKHAH, Mina JALILVAND, Samaneh SARKHOSH, Mina AZARMI, Mohammad MOHSENI

**Affiliations:** 1. Health Management and Economics Research Center, Iran University of Medical Sciences, Tehran, Iran; 2. Dept. of Health Services Management, School of Health Management and Information Sciences, Iran University of Medical Sciences, Tehran, Iran; 3. Health Services Management Research Center, Institute for Futures Studies in Health, Kerman University of Medical Sciences, Kerman, Iran

**Keywords:** Congenital anomalies, Meta-analysis, Prevalence, Iran

## Abstract

**Background:**

Congenital anomalies are considered as main causes of disability and mortality among children in developing and developed countries. Expenditures of hospitalization and treatment procedures for these children impose a large burden on health system and their families. This study aimed to review the prevalence of congenital anomalies among infants in Iran.

**Methods:**

The review of studies was conducted through searching databases including IranMedex, SID, Magiran, Scopus, and PubMed. Descriptive and cross-sectional studies investigating on the prevalence of congenital anomalies among infants were included into the study. Hand search for some related journals and websites was done. The list of studies’ references was reviewed. The data were analyzed using the CMA 2 software.

**Results:**

Of 455 studies, 27 studies were included into the meta-analysis study. The studies were conducted between 1992 and 2014.The overall prevalence of congenital anomalies among infants was estimated to be 2.3%. The overall prevalence rates, in terms of gender, were estimated to be 3% in boys and 2% in girls. While the highest prevalence rates were related to musculoskeletal anomalies (27.5%), skin anomalies (19.7%) and genitourinary system anomalies (15.8%), the lowest prevalence rate was related to respiratory system (1.82%)

**Conclusion:**

The prevalence of congenital anomalies among infants in Iran is high. In order to reduce the rates of these anomalies and complications resulted from them, coping and preventive strategies such as the necessity of genetic counseling particularly in consanguineous marriages seem to be appropriate solutions.

## Introduction

Today’s babies will constitute future young generation and capital in any country depends on healthy and fecund youths, thus their health will guarantee this great capital. Congenital anomalies will be of main factors of ineffectiveness of this great human capital, particularly if they are not diagnosed and treated from the beginning (1, 2). Congenital anomalies are considered as main causes of disability and mortality among children in developing and developed countries. Expenditures of hospitalization and treatment procedures for these children impose a large excess burden on health system and their families (3, 4). This type of anomalies according to definition by WHO includes single or several structural, functional or biochemical and molecular anomalies identified and diagnosable at birth (5). Anomalies based on the intensity are divided into two categories: major and minor. Major anomalies are defined as anatomical anomalies that affect an individual’s life and natural performance. Minor anomalies are referred to structural changes that do not require treatment or can be improved through simple methods (6).

Defects at birth are the main cause of infant mortality; therefore, 21% of mortality during infancy results from these anomalies. This type of anomalies is the fifth leading cause of reduced life expectancy before the age of 65 and is one of the main reasons for disabilities. Two to three percent of newborns have serious structural anomalies at birth and these anomalies are diagnosed in other 2%–3% of infants by the end of the fifth year that constitutes a total of 4%–6% (7). Although various factors have been identified as factors affecting congenital anomalies (including genetic and environmental factors, teratogens such as mother’s addiction to alcohol, diabetes, malnutrition, infection, hyperthermia, drug use and exposure to chemicals or radioactive), 66% of anomalies occur with unknown causes (3, 8). Furthermore, one of health issues in the country is the existence of congenital anomalies and disorders related to consanguineous marriage, which impose a large financial burden on the country and mental and economic burden on family. Those who have these anomalies not only deal with pain and suffering in their life but also impose a large burden of economic and social hardships on their families and society (9).

In general, treatment and rehabilitation of disabled people due to congenital anomalies impose large costs on a society and desired results for person with disability and society are not always obtained. Furthermore, some severe congenital anomalies cause miscarriage or intrauterine fetal death. The identification and prevention of the incidence of congenital anomalies are more cost-effective for societies rather than the treatment or rehabilitation (10) and any serious effort to identify factors affecting congenital anomalies and to prevent them will lead to make the more health and well-being for future generation and to avoid socio-economic harms (3).

The prevalence of the more congenital anomalies in infants born alive throughout the country imposes mental and physical pain and suffering on these patients and their families; increases their treatment costs in various ages of their life, and decreases quantity of productive human resources in the society. The current study aimed to review the prevalence of congenital anomalies in infants.

## Methods

The reviewed documents consist of descriptive and cross-sectional studies selected through searching databases including IranMedex, SID, Magiran, Scopus, and Pubmed, with no time limit. The method of searching studies was mainly done using valid keywords related to prevalence, anomaly and congenital. In the next step, the identified studies were reviewed and appropriate studies were selected based on inclusion criteria.

The inclusion criteria in this study included descriptive and cross-sectional studies on the prevalence of congenital anomalies among infants in Iran; studies were in English and Persian, with no time limit. The exclusion criteria included those studies that mentioned just one specific congenital anomaly (such as the prevalence of Clubfoot congenital deformity); studies that investigated the prevalence of congenital anomalies in infants among a specific group of mothers (such as mothers suffering from Insulin-dependent diabetes); qualitative studies, studies presented in conferences; and interventional studies.

The search in any database was done through searching titles and abstracts in a way that all studies and abstracts in which the words congenital anomaly and factors affecting anomaly were mentioned in their titles, were selected. Then the titles of all studies searched the databases were listed. The list of selected studies was screened in order to identify the most appropriate ones to the issue and studies with repeated titles were excluded. The reference management software (Endnote X6) was used to organize, remove duplicates and assess titles of abstracts.

Finally, two authors reviewed the abstracts and full-texts of studies based on the inclusion criteria. The STROBE checklist (11, 12) was used to assess the quality of the identified studies and the studies of high quality were selected.

For meta-analysis, meta-analysis statistical techniques were used to calculate the overall prevalence and the data were analyzed using the CMA: 2 software. The I^2^ index was used to test the heterogeneity of the studies; since the difference of prevalence rates in various studies was high (heterogeneity of the studies), the random effect model with 95% confidence interval was used.

## Results

**Fig. 1:**
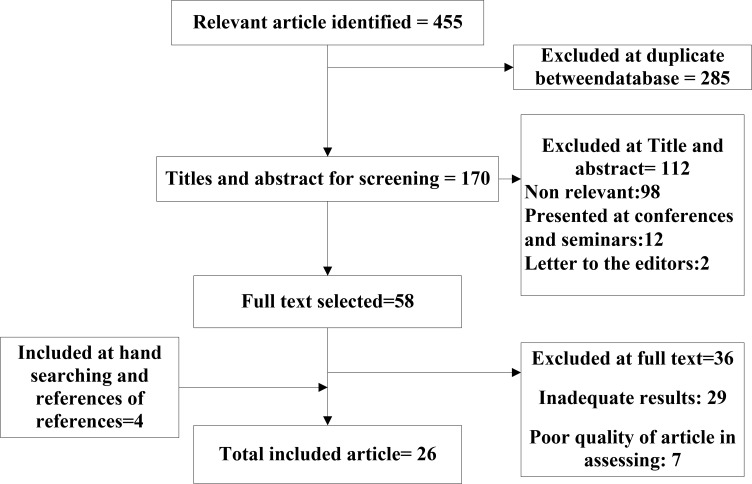
Bibliographical searches and inclusion process

Twenty-six studies were selected for meta-analysis (Table 1). The studies were conducted between 1992 and 2014 in Iran. Overall, 215756 infants had been investigated in the total of studies. The results showed that the prevalence of congenital anomalies among infants varied from 0.4% in Babol to 5.5% in Zanjan, Iran. The overall prevalence in Iran, among boys, and the girls were estimated to be 2.3%, 3%, and 2%, respectively. The highest prevalence rates of anomalies respectively were related to musculoskeletal anomalies (27.5%), skin anomalies (19.7%) and genitourinary system anomalies (15.8%). The lowest prevalence of anomalies was related to respiratory system (1.82%). The ratio of anomalies in boys (58.3%) was higher than in girls (41.1%). Fig. 1 shows the flowchart for the identification of studies.

**Table 1:** Main characteristics of included studies

**Author, Year**	**Setting**	**Sample**	**Sample Size**	**Method**	**Prevalence of Anomaly (%)**	**The ratio of anomaly between boys and girls**	**Type of Anomaly (%)**
Shajari, H et al: 2006(31)	Shariati Hospital, Tehran	All of the newborn that were born during three years	3840	Retrospective	3.1	Boys: 56.8 Girls: 39.8	Skeletal(41.9), Central Nervous (16.2), Head & neck(12), Genitourinary(12), Digestive(3.4), Hearing(1.7), Dermal(0.9), Visual system(0.9), Combine(11.1)
Akbarzadeh, R et al:2008(7)	Mobini Hospital, Sabzevar	All live born infants From first bahman 2005 to first bahman 2006	7786	Descriptive, Cross-sectional	2.4	Boys: 56.5 Girls: 43.5	Musculoskeletal(43.97), Genitourinary(17.78) Cardiovascular(6.8), Neurological disorders (14.7) Chromosomal anomalies (5.75), Digestive system (6.8) Multi anomaly(4.2)
Hematyar M, Khajooei P : 2003(6)	Javaheri Hospital, Tehran	1000 live born infants from farvardin to shahrivar 2003 that inter to the hospital by consecutive	1000	Cross-sectional	5.2	Boys:62 Girls:38	Musculoskeletal(42), Genitourinary (38), Cardiovascular (4), Central nervous (4), Digestive system (4), Other anomalies (4)
Nazemi Gheshmi, AM et al:2007(16)	Two maternity, Bandarabbas	Available sampling from 7007 sample.	7007	Descriptive, Cross-sectional	3	Boys:66 Girls: 33.4	Skull (6.2), Face (4.3), Hair (2.4), Lips and mouth (5.6), Aye(6.2), Ear (5.6), Neck(3.3), Chest, back, nipples, abdomen (7.6), Skin(6.7), Hand(6.2), Leg (10.5), Reproductive organs (35.4)
Hosseini, S et al:2014(32)	Amiralmomenin Hospital, Sistan	All infants born alive and the congenital Anomalies were detected from 1800 infants in three months	1800	Descriptive, Cross-sectional, Analysis	1.8	Boys:55.8 Girls:	Spinal cord (23.5), formation of organs (20.5), Abnormalities in the abdomen (5.8), Abnormalities in the ear, throat and nose and face (29.5), Genitourinary(11.8), Chromosomal anomalies (8.9)
Anvar ahmadi, M & Shah mohammadi, F: 1997(33)	Taleghani hospital of Arak	Live birth in Taleghani hospital, Arak in this year.	2510		1.4	Boys:1.34 Girls:0.72	Skeletal
Khosravi, Sh:2000(34)	Taleghani & Ghods hospital of Arak	All of the newborns that were during spring 2000	2069	Descriptive, Cross-sectional	0.82	Boys:41.18 Girls:58.82	.77.47 % of newborns were with 1 Anomalies that Disorders were in central nervous system, head and neck and skeletal Respectively
Zamani, A et al:2000(35)	Emam Khomeini & Shariati Hospital	All of the newborns in Emam Khomeini & Shariati Hospital were examined in the first 24 h.	4073		Entirely 4073 newborn were involved in this study That 150 case had at least 1 minor or major anomaly	Boys:48.7 Girls:46	
Jalali, H et al:2011(5)	Rasht hospitals	All of the newborn that were born during the year of 2011	1824	Discriptive-analysis	4.2	Boys:4.8 Girls:3.6	Skeletal (37.7), central nervous (10.4), Ear, face and neck (1.3) Genital: (16.9), Urinary(13), Digestive(2.6), Cardiovascular : (13), Respiratory:(1.3), Other syndrome (3.9)
Mahdi nasab, H et al : 2006–2007(1)	Imam khomeini and Razi hospital, Ahvaz	All live birth with a gestational age of 28–42 wk were evaluated for congenital abnormalities of the upper and lower limbs.	5087	Prospective	1.69		Congenital clubfoot(13.96), Hip dysplasia (11.63), Hand polydactyly(3.49), metatarsus adductus (2.33), bilateral hand and foot (1.16), polyductyly (1.16), hand syndactyly (1.16) and vertical talus(1.16)
Hajian, K et al : 2001(36)	Shahid yahyanejad hospital, Babol	All of the newborn that were born during the year of 2001	3756	Cross-sectional study	0.4	Boys:0.5 Girls :0.3	
Ketabchi, H et al: 1992–1993(37)	Mirza koochak khan and Akbarabadi hospitals, tehran		23160	Descriptive	0.47		Anansephaly(0.17), Espina biphida (0.014), Hydrosephaly(0.14), Dermal sinus(2.8), Ansphalosel (5.5 of nervous, Microsephaly (6.3) Ceraniosynustosis (2.8)
Shokouhi, M & Kashani, KH: 1999 (38)	Fatemiyeh hospital, Hamedan	All of the newborn that were born during the first 6 month of 1999.	4252	Descriptive	2.8	Boys:64.7 Girls:35.3	Genitourinary (48.7), Skeletal (23.1), Head and face (8.5) Dermal(6.8), Central Nervous (2.6)
Marzban, H et al :2000–2001(13)	Valiasr hospital, Zanjan	All of the newborn that were born during these years and be examined at least 1 time by children assistant.	2345	Descriptive	5.5	Boys:7.2 Girls:3.8	Genitourinary (2.7), Skeletal (2), Nervous (0.7), Mouth and face: (0.5), Preneh (0.2)
Alijahan, H et al : 2011(3)	Ardebil hospitals	All of the newborn that were born during azar 89 to mordad 90.	6868	Cross-sectional	0.82		Skeletal (35.1), Central nervous (22.8), Digestive: (17.5) Genitourinary (15.8), Chromosome: (8.8)
Kaviani, H et al: 2007–2010(39)	13 Hospitals, Golestan	All live birth in 13 hospitals with anomaly in Golestan	92420	Descriptive-analysis	_ Gorgan: 20.46 _ Cord-kooy:12.53 _ Aliabad:10.86 _ Gonbad: 8.92 In west: 9.3 _ in center: 20.46 _ in east: 8.79		Cardiovascular:(52.37)
Ebrahimi, S : 1999(40)	Maternit of Shahid beheshtiy, Yasouj	Live birth in Maternit of shahid beheshtiy, Yasouj	1317		3.9	Anomaly in boys: 3.7 times girls	Nervous system (13.8)
Abedirad, I et al:2008(41)	Motahhari hospital, Azarbaijan	All birth records (live birds and stillbirds) from January 2001 through June 2005	14121	Cross-Sectional	Total: 1.87	Boys: 50.5 Girls:49.4	Central nervous system (52.65), Musculoskeletal system (23.86), Digestive system(7.20), Urogenital system (6.82), Ear and neck(4.17), Chromosomal anomalies(4.92), Conjoined twins(0.38)
Golalipour M.J, Et al: 2005(10)	Dezyani hospital, Gorgan	The newborn and stillborn babies delivered during a 20-month period, January 1998 to August1999	10 000	Cross-sectional Descriptive Analytical	1.01	Boys: 1.19 Girls:0.76	Musculoskeletal system (0.047), Central nervous system (0.036), Genitourinary system (0.026), Digestive system (0.017) Eye, ear, face and neck (0.006), Oral/cleft lip and palate (0.014), Chromosomal anomalies (0.006), Other anomalies (0.004)
Tootoonchi, P: 2003 (42)	Four teaching hospitals In the south of Tehran	All of the newborns were Examined by a pediatrician during the first 24 h of life From September 1999 until March 2000	2291	Cross-sectional	2.41		Central nervous system(0.69), Eye, Ear, Face and Neck(0.48) Circulatory system(0.08), Respiratory system(0.17), Cleft lip and Cleft palate(0.22), Other congenital anomalies of Digestive system(0.08), Genital system(1.06), Musculoskeletal system(1.32), Chromosomal abnormalities(0.04)
Akhavan Karbasi, S et al :2007(43)	The maternity hospitals in Yazd	All of the new-borns and stillborn babies delivered at all maternity hospitals in yazd during 8 month period, from October 2003 To June 2004	4800	Cross-sectional	2.83	Boys: 2.86 Girls:2.68	Muscloskeletal system(33.18), Central nervous system(11.81), Genitourinary system(11.36), Eye, ear, face and neck(10.90), Cleft lip and Cleft palate(9.54), Digestive system(9.54), Cardiovascular(7.72), Respiratory system(1.81)
Pouladfar, GH. R& Malahzadeh A.R: 2005(44)	A university hospital in Bushehr	All of the newborns from Aug 2002 to Mar 2003.	750	Prospective	5.03	Boys: 5.55 Girls: 4.46	Musculoskeletal system (2.25%), Nose (0.14), Ear (0.42), Mongolian spot (6.01), Hydrocele (7.69), Genitourinary system (1.39), Skin (0.99), Positional clubfoot (1.82), Sacral dimpling (0.56).
Tayebi, N et al :2010(45)	Shahid Sadoughi hospital, Yazd	The newborns who were Born during 9 months period from April to December 2008.	195	Cross-sectional	3.76	Boys: 1.92 Girls:1.58	Clubfoots(0.41), Congenital Dislocation of Hip joint(0.25), Polydactyly(0. 5), Simian crease(0.25), General Nervous system(0.16), Myelomeningocele+/− Hydrocephalus(0.16), Anencephaly(0.1), Microcephaly(0.25), Genitourinary system(0.16), Ambiguos Genitalia(0.1), Polycystic kidney(0.33), Eye, ear, face and neck(0.25), Iris heterchromia(0.25), Preauricular tags(0.1), Cleft lip and cleft palate(0.16), Cleft Lip+/− Cleft Palate(0.33)
Ahmadzadeh, A et al:2008(46)	Arvand hospital in Ahwaz	All of the neonates born at Arvand hospital in Ahwaz from 2004 to 2006 were registere	4660	Prospective	2.02	Boys: 2.38 Girls:1.61	Musculoskeletal, System(0.79), Clubfoot (0. 32), Congenital Dislocation of hip(0.32), Polydactyly(0.09), Syndactyly(0.04), Oligodactyly(0.02) Genitourinary system(0.71), Hypospadias (0.49) Hyronephrosis(0.11), Epispadias(0.07) Polycystic(0.02), kidney disease Microphallus (0.02) Central nervous System(0.24), Meningocele Meningomeylcele(0.11), Microcephaly(0.11), Anencephaly(0.02), Digestive system(0.11) Imperforated anus(0.07), Cleft lip/cleft palate(0.04) Chromosomal Anomalies(0.09), Down’s syndrome(0.09) Cardiovascular System(0.06), Acyanotic heart disease(0.06) Respiratory tract(0.02), Pulmonary Hypoplasia(0.02)
Dastgiri, S et al :2007(47)	Three university-hospitals of Tabriz University of Medical Sciences	All births registered/notified to three university-hospitals of Tabriz University of Medical Sciences, from 2000 to 2004	1574	The cases study	1.65		Nervous system anomalies (0.43), Genito-urinary tract and kidney(0.39), Anomalies of limb (0.26), Chromosomal anomalies (0.0 8), Cleft lip with/without palate (0.10), Congenital heart disease(0. 13), Musculoskeletal and connective tissue anomalies (0.07), Digestive system anomalies (0.09), Eye and ear anomalies (0.03), Other anomalies (0.03)
Masoodpoor, N et al :2008(14)	Niknafs university hospital of Rafsanjan	All of the newborns that were born 2007–2008	6089	Descriptive	2.93	`	
Khoshhalrahdar, F et al:2013(48)	Ganjoyan hospital in Dezfol	All of the newborns that were born 2013	4235	Cross-sectional	3/21	Boys: 3.88 Girls:2.56	Musculoskeletal system(0.97), Cardiovascular System(0.71)

**Fig. 2:**
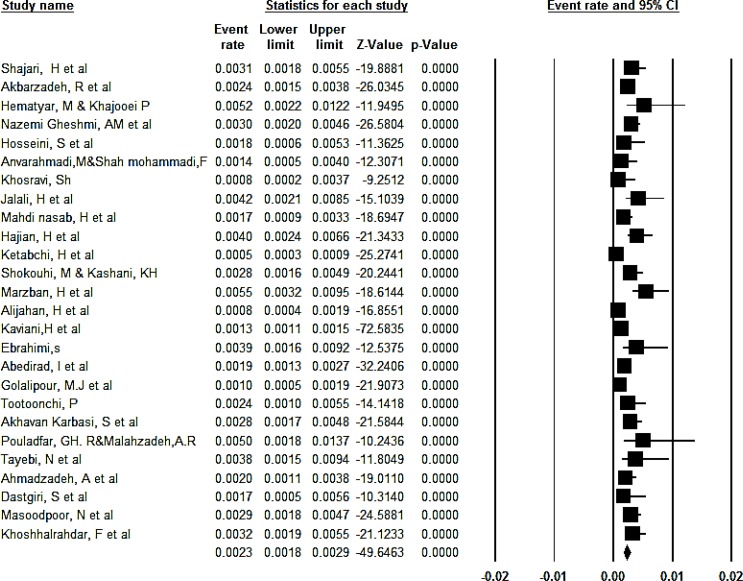
The overall prevalence of congenital anomalies in Iran

**Fig. 3:**
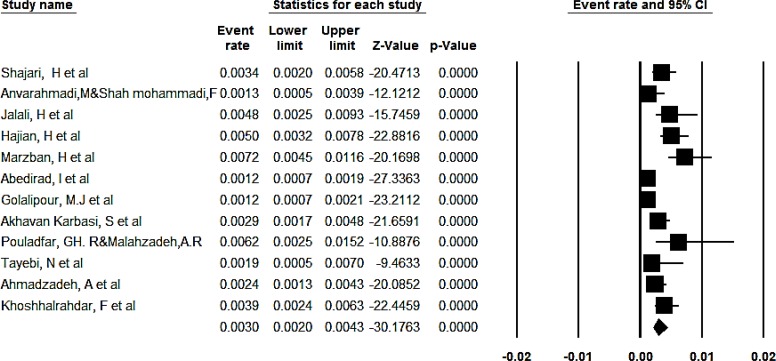
The overall prevalence of congenital anomalies in boys

The overall prevalence of congenital anomalies based on the random effect model was determined to be 2.3% (95% CI, 1.8%–2.9%) (Fig. 2). Totally, 95% CI for the prevalence was drawn for each study in the horizontal line format (Q=109.2, df = 25, *P*<0.001, I^2^= 77.1). The overall prevalence of congenital anomalies in boys based on the random effect model was determined to be 3% (95% CI, 2%–4.3%) (Fig. 3). Totally, 95% CI for the prevalence was drawn for each study in the horizontal line format (Q = 51.6, df = 11, *P*<0.001, I^2^= 78.7).

**Fig. 4:**
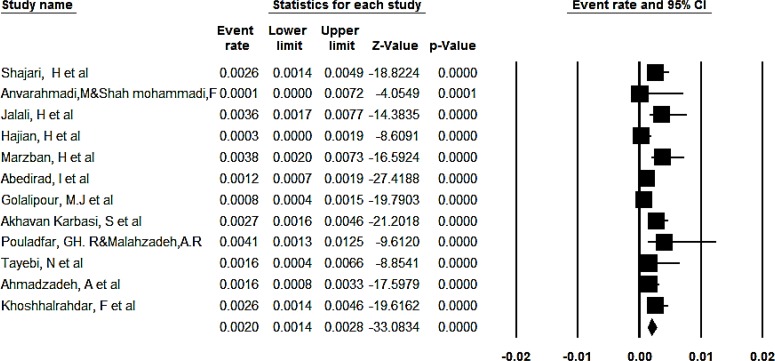
The overall prevalence of congenital anomalies in girls

**Fig. 5:**
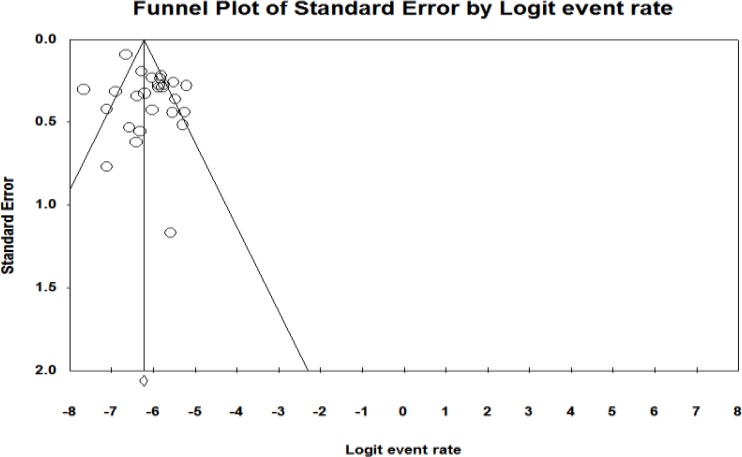
Funnel plot of standard error by event rate

**Fig. 6:**
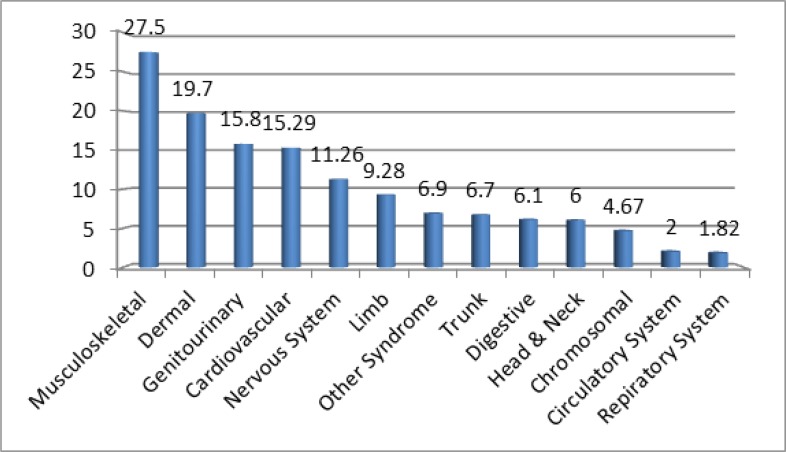
Ratios of anomalies in different body systems among infants

The overall prevalence of congenital anomalies in boys based on the random effect model was determined to be 2% (95% CI, 1.4%–2.8%) (Fig. 4). Overall, 95% CI for the prevalence was drawn for each study in the horizontal line format (Q=28.4, df = 11, *P*<0.001, I^2^= 61.3). The result of funnel plot show there was possibility publication bias among studies (Fig. 5, 6).

## Discussion

The results of this study showed that the overall prevalence of congenital anomalies among infants in Iran is 2.3%; and its prevalence rate was estimated to be higher in boy infants (3%), than girl infants (2%). The highest prevalence rates of anomalies respectively were related to musculoskeletal anomalies (27.5%), skin anomalies (19.7%) and genitourinary system anomalies (15.8%), while the lowest prevalence of anomalies was related to respiratory system (1.82%).

The prevalence rates of anomalies are different among the studied populations and in various body systems and organs. Genitourinary system anomalies were followed by musculoskeletal anomalies as the most prevalent anomalies (13). However, findings of the studies (7, 14), similar to the current study, show that the most prevalent anomaly is related to musculoskeletal system.

In addition, in Bahrain, the highest prevalence rates of anomaly are related to musculoskeletal system diseases (2.28) and genitourinary system diseases (2.13) (15). Through a simple review of the documents, the reason for such a difference in the prevalence of all types of congenital diseases in reports of different studies can be the difference in the prevalence of serious congenital anomalies among various races which environmental factors sometimes have impact on their incidence (16).

The percentage of the prevalence of congenital anomalies among infants in Iran (2.3%) is lower than that of some studies conducted in other countries in the world. The rates of congenital anomalies were reported to be 7.6% in Uganda (17) and 3.17% in Egypt (18). The prevalence rates of congenital anomalies were reported as following: 2.7% in nine countries in Latin America (19), 2.39% in 22 European countries (20), 2.7% in Bahrain (15), 1.28% and 2.11% in two studies in India (21, 22) and 2.07% in Turkey (23) which is close to the estimated prevalence rate of anomaly in Iran. However, the prevalence rates of anomalies in Lebanon (1.65%) (24) and in China (1.54%) (25) are lower than the estimations in Iran. The difference in the prevalence rates of congenital anomalies in different parts of the country and the world can be due to differences in genetic, racial, cultural and socio-economic factors among individuals and the assessment method of infants. The difference in methods used for diagnosing anomalies and characteristics of studied populations (live or dead babies) can be a reason for part of this difference (26–28).

There is relationship between many congenital anomalies and gender of infant; and the relationship between each of these anomalies and gender of infant should be considered in further larger studies (29). In the current study, the prevalence of anomalies among boys (58.3%) was higher than among girls (41.1%). Suffering from congenital anomalies in a study conducted in the Czech Republic in 2000 and also in a study in 1980, similar to the results of the current study, was higher among boy infants than girl infants (30).

In order to prevent the birth of infants with severe congenital anomalies and given the necessity of healthy, productive and effective future generation of human resources in the society, the necessity of genetic counseling especially in consanguineous marriage and care initiatives for pregnant mothers is of particular importance.

## Conclusion

The prevalence of congenital anomalies in different body systems in infants is high. Even if infants born with these anomalies can survive for years, they will live all their lifelong with complications and hardships due to these diseases. Moreover, their families also face heavy cost incurred on improving and reducing effects of these anomalies in their children. Thus, in order to prevent the birth of such infants the necessity of genetic counseling especially in consanguineous marriage is recommended. Furthermore, caring before, during and after pregnancy including healthy nutrition, proper exercise, mother’s lack of exposure to dangerous rays and radiations and avoiding stress and anxiety can play a useful role in the birth of healthy and normal infants.

## Ethical considerations

Ethical issues (Including plagiarism, informed consent, misconduct, data fabrication and/or falsification, double publication and/or submission, redundancy, etc.) have been completely observed by the authors.
